# Covid-19 and the 2020 presidential election

**DOI:** 10.1007/s10602-022-09371-z

**Published:** 2022-10-31

**Authors:** David M. Mitchell

**Affiliations:** Missouri State University, Springfield, MO United States

**Keywords:** COVID-19, Elections, Economic performance, Voter turnout, D72, E24, H12, I18, R23

## Abstract

Whether deserved on not, US Presidents often receive the blame or the credit for the nature of the economy and direction of the country. Therefore, the status of the economy and the country in an election year can be a very important factor in election success for an incumbent President (or his party if an incumbent is not running). This is especially true in ‘battleground states’ due to the presence of the Electoral College system where Presidential candidates need only win different combinations of states in order to become President. However, the 2020 Presidential election was vastly different from past election cycles in that an additional variable, COVID-19, was added to the decision calculus of voters. Eventually, the 2020 election came down to the extremely slim margins in three states (Arizona, Georgia, and Wisconsin) and thin margins in two others (Pennsylvania and Michigan). This paper shows that deaths from COVID-19 at the county level played a small role in demotivating voters to turnout in 2020 to cast their vote for Joe Biden as President. In other words, without Covid-19, President Trump’s losses within these five states would have been even larger.

## Introduction

In 1992, a political strategist named James Carville worked on Bill Clinton’s presidential election campaign and famously stated, ‘It’s the economy, stupid’, as a reminder to both Bill Clinton and his campaign staff to focus on the economy as a means of winning the election. In many ways this advice is still repeated every four years during the US presidential election cycles. In December of 2019, economic conditions, despite voter sentiment about Donald Trump as a person, looked favorable towards then President Trump winning re-election in 2020. In a poll done by SSRS research and released by CNN on December 20, 2019 53% of respondents had an unfavorable opinion of Donald Trump but fully 76% of respondents rated the economy as good while only 22% rated it poorly.^1^ Similarly, a USA Today/Suffolk University poll released at the same time predicted that Donald Trump would win re-election by 3 to 10% points over hypothetical match-ups between Joseph Biden, Pete Buttigieg, Bernie Sanders, Michael Bloomberg, and Elizabeth Warren.^2^

Although ‘pocketbook issues’ can be one of the main drivers of voters’ decisions of whether to vote and who to vote for, it is possible that other factors such as war, pandemics, or political corruption scandals can rise to the top of voters’ concerns and supplant economic issues as the main driver in voters’ minds. The emergence of Covid-19 in late 2019 quickly changed economic and political conditions not only within the US, but worldwide. Groshen (2020) found that by September of 2020, Covid-19 had produced a deep and abrupt shock to the economy and that the pace of recovery has slowed significantly since June of 2020. Forsythe et al., (2020) also found that the economy had faced broad-based collapse and that it would take more than simply lifting stay-at-home orders to restore the economy. Furthermore, Covid-19 produced drastic reductions in both the scope and structure of global trade (Vidya & Prabheesh, 2020).

However, Covid-19’s impact was not just economic, but political and social as well. As of November of 2020, worldwide deaths from Covid-19 were 1.226 million with the US just shy of 260,000. Some researchers have attempted to place the blame for these higher death rates in the US on President Trump and his lack of leadership during the pandemic (Adolph, 2020) while others noted that lockdowns and quarantines have been correlated with increased domestic violence, loneliness, and boredom which has damaged people’s mental health (Brodeur et al., 2021; Bullinger et al., 2021). Finally, widespread protests against public health actions such as lockdowns and quarantines were seen across the United States and the world. Many protestors came to view these public health actions as a violation of individual freedom and examples of government overreach. The confluence of a global pandemic, the national, state, and local responses to it, changes in economic conditions, and other factors promised to possibly alter the decision calculus of voters in November of 2020.

Nevertheless, due to the unique nature of Presidential elections in the United States vis-à-vis the electoral college, the convergence of these factors on voter behavior was likely to be most impactful in only a few ‘battleground states’ (Cain et al., 2007). To illustrate this, consider that in 2020 twelve states received 96% of all of the general-election campaign events. This institutional structure means that the entire US Presidential election can come down to the difference of just a few votes within just a few states. Ensuring election wins within the battleground states is crucial in winning the election overall.

By the time the 2020 Presidential election was over, Joseph Biden had won both the popular vote and the electoral college vote. However, throughout the election cycle and after the election results were published, President Trump continued to proclaim that the election was stolen. He and his attorneys focused on 5 different states: Arizona, Georgia, Michigan, Pennsylvania, and Wisconsin. These five states were won by President Trump during the 2016 election but had flipped to Biden in 2020. Georgia was lost to President Trump by only 11,779 votes or just 0.24% of the total votes cast in the state for all presidential candidates. Similarly, Arizona had been flipped by only 10,457 votes (0.31%) while Wisconsin flipped with 20,682 votes (0.63%). Results in Pennsylvania and Michigan, while still close by election standards, were firmer. President Trump has lost Pennsylvania by 80,555 votes (1.16%) and Michigan by 154,188 votes (2.78%).

This paper seeks to analyze the election results within these 5 states and to measure what impact Covid-19 had on changing voters’ minds relative to the 2016 election. The inclusion of Covid-19 as a possible factor in altering election results is a unique and novel difference from the past literature on election cycles. A brief literature review of the impact that different economic and political conditions have on election results follows. The data and model used in this paper is then reviewed followed by a discussion of the results and a brief conclusion as well as areas of future research.

## Literature Review

Election results and the different factors that can impact them has been studied in detail for many years, but the conclusions of the literature are far from certain. The existence or nonexistence of a theory of ‘political business cycles’ was first examined by Hibbs (1977, 1986), Nordhaus (1975), and McRae (1977) and most recently by Grier (2008). Nordhaus and McRae believe that incumbent politicians would attempt to alter the course of the macroeconomy to increase their chance of reelection and Grier finds evidence of the existence of a political business cycle within the US as recently as 2004. Fair (1978, 1982) found that economic conditions could alter presidential elections but had results that were in direct contrast to Kramer (1971). More recent scholarship though confirmed the importance of economic factors on the voters’ decision for President (Grier, 2008; Becher & Donnelly, 2013; Huberman et al., 2018). An alternative to direct manipulation of the macroeconomy is altering the national government’s budget in a way that is favorable to the incumbent party (Klomp & de Haan, 2013).

Hibbs rebuffed the idea of a political business cycle and instead promoted the concept of a ‘partisan theory’. In partisan theory different political parties, Republican and Democrat, attempt to change the set of economic conditions which they believe align most closely with their constituents’ desires. It is generally thought that Republican politicians focus on controlling inflation while Democrat politicians concentrate on reducing unemployment (Smyth and Taylor, 1992). Furthermore, there appears to be evidence that Republican candidates are punished more for higher unemployment than Democrats (Burden & Wichowsky, 2014; Mitchell & Willett, 2006).

There are of course factors other than national economic performance that determine election success in both the US and abroad. Collier & Hoeffler (2015) find that incumbency itself is an important determinant while other factors that have been examined for their influence on elections include war, industry employment mix, populism, economic insecurity, and partisanship. Stevens (2015) observes that right leaning candidates are less likely to get punished by voters for entering or continuing wars relative to left leaning candidates whereas Jensen et al., (2017) observed that counties with higher concentrations of employment in industries that are helped by international trade are more likely to vote to retain incumbent candidates who are seen as being ‘pro-trade’. The opposite occurs in counties with high concentrations of employment in industries hurt by globalization. This bifurcation effect can snowball as the regions of a country in economic decline becomes more likely to move towards populist and other extremist candidates who promise to reverse the economic malaise that they face, restore social conditions, or alter immigration polices (Margalit, 2019; Boleslavsky & Cotton, 2015; Burden & Wichowsky, 2014; Colantone & Stanig, 2019; Wang, 2021).

It is also possible, at least in the US, that Presidential elections are becoming closer over time due to an increasingly divided and hyper partisan electorate. The average winning margin in US Presidential elections has fallen steadily from 12.5% in the 1950 and 1960s to 3.5% in recent elections. Having an increasingly rigid and polarized electorate heightens the need for increased voter turnout of voters loyal to a particular party in order to win an election (Abramowitz, 2014; Martins and Veiga, 2013; Charles & Stephens 2013). Over the past nine US Presidential elections, no one has won by a 10-point lead in the popular vote which is generally considered to be a ‘wide margin’. There is some evidence that this hyper partisanship is being driven by open versus closed primaries within the different states as well as the timing of the primary relative to the party’s nominating conventions (Calcagno & Westley, 2008).

Covid-19’s large worldwide impact on social and economic structures promises to become an additional factor for researchers to examine for its effects on many facets of society including elections. If voters hold political leaders such as President Trump or their local governor ‘responsible’ for covid deaths and cases, this would likely have a negative effect on that leader’s ability to win an election; however, if they are not held ‘responsible’ by voters, it is possible that Covid-19 could benefit or have no effect on the election. There are three primary ways that Covid-19 deaths or cases could have impacted election results. The first way is the *alteration effect* whereby voters who had intended to vote in the 2020 election before Covid-19 change the party of their previously intended vote. Of course, this change of vote can be either positive or negative for an incumbent. The second transmission effect is the *mobilization effect* in which Covid-19 mobilizes or demobilizes voter blocks to come to the polls or stay home. Once again, this effect can be either positive or negative for the incumbent.

The final transmission effect is the *obituary effect*. Since Covid-19 has an unequal effect on people based upon age and other comorbidities, it is possible that Covid-19 tends to kill more of one political party’s supporters than another. In short, if Covid-19 kills persons over the age of 65 in a greater proportion than the general population, and if people over the age of 65 vote in a distinctively different way than younger people, than it’s possible that Covid-19 could impact election results simply because it kills off potential voters sympathetic to one party or the other. It bears repeating for the reader that the total effect of Covid-19 on voting behavior in the 2020 Presidential election need not be large in overall size to impact the election—it just needed to be large enough to overcome razor thin statewide vote totals in a few battleground states. Although future research can tease out what effect Covid-19 had on voting behavior in other states and political races, it is unlikely that covid’s effect would have been large enough to change the final statewide results for President in states such as California or Wyoming.

It is the combination of these transmission effects that necessitates looking at Covid-19’s impact at the county level instead of aggregating at the state or national level. Furthermore, although Covid-19’s overall aggregate impacts on the nation might have been well known, it is its local effects that drive economic and social changes at the local level, e.g. the extent of masking ordinances, reduced hours at places of business, etc. Voters in relatively rural counties that were only lightly touched by Covid-19 might assign little impact from it into their voting decision while voters in heavily urbanized counties that experienced severe impacts from Covid-19 would be more likely to assign greater weight to it in their voting calculus.

## Data, Theory, and Model Structure

County level data for 2016 and 2020 was collected for each county in the states of Arizona, Georgia, Michigan, Pennsylvania, and Wisconsin. The 2020 election within these five states is unusual not only because these states were the only states to flip on a close vote count from the 2016 election, but for other reasons as well. Only one county out of all of the counties in these five different states experienced a decrease in the number of total votes cast between the 2020 and 2016 election [Stephens County, GA, -94 voters]. Secondly, in only two counties out of all of the counties in the five different states did President Trump see a decrease in his raw county vote total between the 2020 and 2016 election [Rockdale County, GA, -466 votes; Stewart County, GA, -4 votes]. Thirdly, in only one county out of all of the counties in the five different states did the Democratic Presidential candidate experience a decrease in the raw vote total between the 2020 and 2016 election [Stewart County, Georgia, -40 votes].

Data on each county’s population over the age of 18 and over the age of 65 was collected for 2016 and 2020. Other demographic data included the percentage of the county’s population that is White and not Hispanic. Economic data consisted of the county unemployment rate in October of 2020 and October of 2016 and per capita county income from 2016 to 2020.

Election result data for each of the five states consisted of the number of registered voters, total votes cast, and total votes received by Clinton, Trump, and Biden in 2016 and 2020. Finally, county level data on the number of deaths attributed to Covid-19 as well as the number of cases on each day was assembled from the CDC Covid-19 Data Tracker. County level data on cases and deaths was then examined relative to national data in several different configurations since Covid-19 is more likely to kill persons over the age of 65 than the general population. On election day, national data showed a death rate of 90.487 deaths and a case rate of 3,683.76 cases per 100,000 persons if one examined total deaths and cases over the entire population over age 18. Focusing on just the population over the age of 65, these national totals equate to a death and case rate of 429.67 deaths and 17,492.08 cases per 100,000 persons. These national case and death rates, as well as the growth rate in the case and death rates from either September 1 or October 1 to election day, were compared to equivalent county level data. Table 1 lists the variables analyzed with definitions while Table 2 conveys descriptive statistics. Note that not all variables listed in Tables 1 and 2 are used in the model analysis. Some variables are listed simply for reader information and interest. There was a total of 396 observations.

As noted earlier, in an era of a hyper partisan electorate, voter turnout, as opposed to trying to change voter opinions, can be the key to winning an election. These 2020 levels of partisanship make voter turnout one of the most important, if not the most important, elements in winning an election. The consequence of voter turnout is confirmed by a comparison analysis at the county level of the 2016/2020 election compared to the 2008/2012 election in Table 3. Both of these elections featured an incumbent, albeit of different parties. However, between the 2008 and 2012 elections, 283 (71%) of the counties in these five states saw a decrease in the number of voters while 113 (29%) of the counties saw more voters in 2012 versus 2008. Comparisons of raw voter turnout in other past presidential elections confirms this pattern of increases in voter turnout in some counties and decreases in other counties. However, this pattern does not hold for the 2016/2020 election comparison. Every county except one saw an increase in the number of voters in 2020 relative to 2016. The razor thin loss by Trump in the 2020 election demonstrates how important voter turnout was in creating the election results.

**Table 1:** List of Variables and Definitions

Variable	Definition
2016POP18	County Population over 18 in 2016
2020POP18	County Population over 18 in 2020
2016POP65	County Population over 65 in 2016
2020POP65	County Population over 65 in 2020
WHITEPOP2016	Percent of County Pop that is White in 2016
WHITEPOP2020	Percent of County Pop that is White in 2020
COLLEGE	Percent of County Pop over Age 25 with a College Degree
POPDENSITY	Population per square mile in 2020
POP65PCT2020	Percent of County Population that is 65 and over in 2020
INCOME2016	County Per Capita Personal Income in 2016 in thds
INCOME2020	County Per Capita Personal Income in 2020 in thds
INCOMEGROW	Growth Rate of Per Capita Income between 2016 and 2020
UNEMPLOY2016	County Unemployment Rate in October of 2016
UNEMPLOY2020	County Unemployment Rate in October of 2020
COVIDCASESPOP18	Number of Total Covid County Cases in 2020 through November election (cases/100k population over 18)
COVIDCASESPOP65	Number of Total Covid County Cases in 2020 through November election (cases/100k population over 65)
COVIDDEATHSPOP18	Number of Total Covid County Deaths in 2020 through November election (deaths/100k population over 18)
COVIDDEATHSPOP65	Number of Total Covid County Deaths in 2020 through November election (deaths/100k population over 65)
COVIDCASESGROW	Percentage Growth in Covid Cases from either September 1st (SEP) or October 1st (OCT) through Election Day
COVIDDEATHSGROW	Percentage Growth in Covid Deaths from either September 1st (SEP) or October 1st (OCT) through Election Day
USCASESGROW	Growth in County Covid Cases from either September 1st (SEP) or October 1st (OCT) through Election Day / Growth in US Covid Cases over the same time frame
USDEATHSGROW	Growth in County Covid Deaths from either September 1st (SEP) or October 1st (OCT) through Election Day / Growth in US Covid Deaths over the same time frame
USCASESPOP18	Number of Total Covid County Cases in 2020 through November election (cases/100k population over 18) minus US Total Covid Cases in same time frame (cases/100k population over 18)
USCASESPOP65	Number of Total Covid County Cases in 2020 through November election (cases/100k population over 65) minus US Total Covid Cases in same time frame (cases/100k population over 65)
USCASERATIOPOP18	Ratio of Total County Covid Cases (cases/100k population over 18) to Total US Covid Cases (cases/100k population over 18)
USCASERATIOPOP65	Ratio of Total County Covid Cases (cases/100k population over 65) to Total US Covid Cases (cases/100k population over 65)
USDEATHSPOP18	Number of Total Covid County Deaths in 2020 through November election (deaths/100k population over 18) minus US Total Covid Deaths in same time frame (deaths/100k population over 18)
Continued on next page	
USDEATHSPOP65	Number of Total Covid County Deaths in 2020 through the November election (deaths/100k population over 65) minus US Total Covid Deaths in same time frame (deaths/100k population over 65)
USDEATHRATIOPOP18	Ratio of Total County Covid Deaths (deaths/100k population over 18) to Total US Covid Deaths (deaths/100k population over 18)
USDEATHRATIOPOP65	Ratio of Total County Covid Deaths (deaths/100k population over 65) to Total US Covid Deaths (deaths/100k population over 65)
VOTERS2016	Number of Voters in a County for the 2016 Election in November
VOTERS2020	Number of Voters in a County for the 2020 Election in November
VOTERGROW	Growth Rate of the number of Voters in a County from 2016 to 2020
REGISTER2016	Number of Registered Voters in a County as of 2016 Election Day
REGISTER2020	Number of Registered Voters in a County as of 2020 Election Day
REGISTERGROW	Growth Rate of Registered Voters in a County from 2016 to 2020
TRUMP2016PCT	Percent of Votes Cast for Trump in 2016
TRUMP2020PCT	Percent of Votes Cast for Trump in 2020
CLINTON2016PCT	Percent of Votes Cast for Clinton in 2016
BIDEN2020PCT	Percent of Votes Cast for Biden in 2020
TRUMPVOTECHANGE	Change in total number of votes cast for Trump within a county (2020 votes – 2016 votes)
VOTESCHANGE	Change in total number of voters within a county (VOTERS2020 – VOTERS2016)
TRUMPVOTECHANGEPCT	Percent of ‘new’ votes received by Trump in 2020 (TRUMPVOTECHANGE/VOTESCHANGE)
VISITTRUMP	Number of times Trump visited a county from Sept 1st to Election Day
VISITBIDEN	Number of times Biden visited a county from Sept 1st to Election Day
VISITPENCE	Number of times Pence visited a county from Sept 1st to Election Day
VISITHARRIS	Number of times Harris visited a county from Sept 1st to Election Day

**Table 2:** Descriptive Statistics

Variable	Minimum	Maximum	Average	SD
2016POP18	1,329	3,211,784	89,513	223,980
2020POP18	1,294	3,380,310	92,163	233,154
2016POP65	394	614,826	18,376	43,033
2020POP65	443	680,876	20,221	47,469
WHITEPOP2016	11.4	98.1	81.6	17.8
WHITEPOP2020	12.0	97.9	81.0	17.8
COLLEGE	3.9	34.7	13.3	5.4
POPDENSITY	0.3	11,022.7	231.7	693.7
POP65PCT2020	5.9	48.2	25.1	5.6
INCOME2016 (thds)	18.2	77.4	38.6	8.4
INCOME2020 (thds)	21.1	95.6	46.2	9.9
INCOMEGROW	3.8	34.5	19.9	4.1
UNEMPLOY2016	2.5	19.5	5.4	1.8
UNEMPLOY2020	2.2	15.5	5.0	1.8
COVIDCASESPOP18	246.6	21,747.4	3,911.6	2,373.6
COVIDCASESPOP65	846.4	346,603.8	17,426.8	19,724.0
COVIDDEATHSPOP18	0.0	627.2	89.2	89.8
COVIDDEATHSPOP65	0.0	2,233.3	376.6	377.2
COVIDCASESGROW_SEP	8.8	1,725.0	190.3	252.5
COVIDCASESGROW_OCT	7.1	604.8	74.1	74.1
COVIDDEATHSGROW_SEP	0.0	2,600.0	123.8	274.0
COVIDDEATHSGROW_OCT	0.0	3,200.0	66.1	204.5
USCASESGROW_SEP	0.2	31.1	3.4	4.5
USCASESGROW_OCT	0.2	20.5	2.5	2.5
USDEATHSGROW_SEP	0.0	99.9	4.8	10.5
USDEATHSGROW_OCT	0.0	266.9	5.5	17.1
USCASESPOP18	-3,437.1	18,063.6	227.8	2,373.6
USCASESPOP65	-16,645.7	329,111.7	-65.3	19,724.0
USCASERATIOPOP18	0.07	5.9	1.1	0.6
USCASERATIOPOP65	0.04	19.8	1.0	1.1
USDEATHSPOP18	-90.5	516.4	-0.9	90.2
USDEATHSPOP65	-429.7	1,803.6	-53.1	377.2
USDEATHRATIOPOP18	0.0	6.7	1.0	1.0
USDEATHRATIOPOP65	0.0	5.2	0.8	0.8
VOTERS2016	904	1,608,875	52,507	125,080
VOTERS2020	928	2,068,144	60,954	149,495
VOTERGROW	-4.5	40.5	15.5	5.9
REGISTER2016	1,157	2,161,716	72,796	176,306
REGISTER2020	1,246	2,595,272	81,696	198,969
REGISTERGROW	-9.1	59.9	14.6	13.6
TRUMP2016PCT	13.47	88.03	59.98	13.32
TRUMP2020PCT	14.08	90.25	61.30	13.60
CLINTON2016PCT	9.72	83.35	35.18	12.91
BIDEN2020PCT	9.02	84.99	37.42	13.55
TRUMPVOTECHANGE	-466	248,304	4,455	13,539
VOTESCHANGE	-94	459,269	8,447	26,584
TRUMPVOTECHANGEPCT	-7.22	175.08	72.01	21.67
VISITTRUMP	0	2	0.08	0.3
VISITBIDEN	0	4	0.07	0.4
VISITPENCE	0	2	0.05	0.2
VISITHARRIS	0	3	0.03	0.2

**Table 3:** Five State Incumbent Election County Results Comparison

County Observations	Obama (2008 to 2012)	Trump (2016 to 2020)
Vote Total Increased	113	395
Vote Total Decreased	283	1
R Vote Increase	274	394
R Vote Decrease	122	2
D Vote Increase	55	395
D Vote Decrease	341	1

Because of the difference in voting patterns displayed in the 2020 election relative to past elections, the standard ‘precent vote share’ for Trump or Biden that might be used in other election related research is not employed here. What follows is a careful explanation of the dependent variable used in this analysis and how it is constructed. Recall that the election in at least three of the five states was won by a very small margin (e.g. 10,457 votes in Arizona and 11,779 votes in Georgia). Margins this small mean that increased voter turnout in a few small counties or just one or two large counties can become crucial to winning the state.

This paper’s model focuses on these ‘new voters’—the ‘increase’ in voters from the 2016 to the 2020 election and employs a similar, but not identical, model to the one used by Charles & Stephens (2013) which examined voter turnout. It is theorized that new Trump voters in the 2020 election in county *C* and state *S* are driven by a vector of demographic, labor force, and Covid-19 pandemic related characteristics which can be represented by Eq. (1)

(1) *TRUMPVOTECHANGEPCT*_CS_ = *β*_*0*_ + *β*_*1*_*D*_*CS*_ + *β*_*2*_*L*_*CS*_ + *β*_*3*_*C*_*CS*_ + *δ*_*s*_ + *e*_*cs*_.

where *D*_*CS*_ represents the vector of demographic and political characteristics such as race, college education, population density, and 2016 election Trump support; *L*_*CS*_ is the vector of labor force characteristics such as income and unemployment rates; and *C*_*CS*_ is the vector of Covid-19 pandemic factors such as case and death counts. State level fixed effects are represented by *δ*_*s*_ and *e*_*cs*_ is the error term. *TRUMPVOTECHANGEPCT* is the percent captured of the number of additional votes received by President Trump at the county level from the 2016 election to the 2020 election.^3^ In this way, the model is focused on the ‘marginal’ voter—i.e., those new votes in 2020. If the variable *TRUMPVOTECHANGEPCT* is larger than 50% for County X, one can conclude that Trump was able to capture a ‘majority’ of these new ‘marginal’ 2020 County X voters. If the variable is positive, but less than 50%, Trump was unable to capture a ‘majority’ of the ‘new’ marginal voters in the 2020 election. If the value is negative, as happens in two counties, Trump not only didn’t ‘gain’ any ‘new’ voters, but also lost some of the voters who voted for him in 2016. Similarly, if the value of the variable is greater than 100%, Biden not only did not ‘gain’ any ‘new’ voters but lost some of the Clinton voters from 2016 to Trump.

To conceptualize how *TRUMPVOTECHANGEPCT* works with real county level data, consider the few counties illustrated in Table 4 where ‘D Votes’ and ‘R Votes’ refers to the total number of votes for the respective party’s presidential candidate in the stated election. ‘R Margin’ is the vote margin, positive or negative, that the Republican Presidential candidate received. Compare two extremes: Bedford County, Pennsylvania and Rockdale County, Georgia. In Rockdale, Republican Presidential candidates are continuing to lose vote share as time progresses. In 2008, John McCain lost the county by 3,335 votes. Four years later Mitt Romney lost the county by 6,307 votes despite the fact that the total number of voters had only increased by 372 persons. In 2016, Trump increased the vote loss to nearly 10,000 with an almost identical number of voters from 2012. Finally, in 2020, Republican candidates lost the county by 18,232. This represents an even more alarming erosion of support for Republicans when one considers that there were only an additional 6,530 voters in the 2020 election relative to the 2012 election. Recall that Trump lost the entire state of Georgia by less than 12,000 votes.

**Table 4:** ‘Marginal Voter’ Example for Five Different Counties

	Apache, AZ	Calhoun, GA	Muskegon, MI	Rockdale, GA	Bedford, PA
*2008 Election*					
Total Votes	24,907	2,212	84,271	37,784	25,787
D Votes	15,390	1,342	53,821	20,256	6,059
R Votes	8,551	862	29,145	16,921	16,124
R Margin	-6,839	-480	-24,676	-3,335	+ 10,065
*2012 Election*					
Total Votes	25,848	2,189	76,163	38,156	21,697
D Votes	17,147	1,298	44,436	22,023	4,788
R Votes	8,250	883	30,884	15,716	16,702
R Margin	-8,897	-415	-13,552	-6,307	+ 11,914
Change in Total Votes	+ 941	-23	-8,108	+ 372	-4,090
Change in R Margin	-2058	+ 65	+ 11,124	-2,972	+ 1,849
*2016 Election*					
Total Votes	28,492	4,062	79,278	38,229	23,637
D Votes	17,083	1,004	37,304	23,255	3,645
R Votes	8,240	2,951	36,127	13,478	19,552
R Margin	-8,843	+ 1,947	-1,177	-9,777	+ 15,907
Change in Total Votes	+ 2,644	+ 1,873	+ 3,115	+ 73	+ 1,940
Change in R Margin	+ 54	+ 2,288	+ 12,375	-3,470	+ 3,993
*2020 Election*					
Total Votes	35,172	6,562	92,444	44,686	27,574
D Votes	23,293	1,103	45,643	31,244	4,367
R Votes	11,442	3,491	45,133	13,012	23,025
R Margin	-11,851	+ 2,388	-510	-18,232	+ 18,658
Change in Total Votes	+ 6,680	+ 2,500	+ 13,166	+ 6,457	+ 3,937
Change in R Margin	-3,008	+ 553	+ 667	-8,455	+ 2,751

If Trump had somehow managed to keep the same vote R and D vote totals in Rockdale County from 2016 to 2020, he would have been almost 3/4th of the way to winning the entire state’s electors.

Bedford County represents a different extreme with Bedford County seeing Republican margins increasing for the past 4 elections. In 2008, John McCain received 10,065 more votes than Barack Obama. By 2012, this lead had grown to almost 12,000 votes despite that fact that there were 4,000 fewer total votes. In 2016 there was an increase of 2,000 voters in Bedford County but President Trump was able to increase his vote count by 4,000 votes. Finally, in 2020 an additional 3,947 voters came out to vote from the previous election, but this time Trump was only able to capture and additional 2,751.

Recall that at the aggregated state level, the benefit of winning lots of small counties by a large margin can be completely offset by losing one or two large counties by a relatively small margin or even outright winning a large county but with a small margin. The average number of voters in a county in 2020 was 60,954; but the minimum number was 928 (Taliaferro County, Ga) while the largest in Georgia was Fulton County at 524,659 and the sample data maximum was over 2 million (Maricopa County, Az). Even if Trump had won all of the votes in Taliaferro County in 2020, this would not be enough to overcome his deficit of -243,904 in Fulton County even though his vote total increased by 19,457 in 2020 over 2016. In other words, generally speaking of course, one still needs to win a few larger urban counties, or at least minimize losses within those larger urban counties, in order to win at the state level.

Visits by Trump, Pence, Biden and Harris are also examined. The literature is somewhat divided on whether visits by candidates helps, hurts, or has no effect on election results. These impacts are usually measured in the form of increased mobilization of one’s voters, and incidentally, counter-mobilization of the other candidates’ voters, and fund raising (Heersink et al., 2021; Abramowitz & Panagopoulos, 2020).

The literature has noticed a growing urban/rural divide where urban areas are more likely to vote democrat and rural areas to vote republican. Therefore, measures of county population size and density are expected to exhibit a negative effect as a county’s population grows larger and denser. Similar to this urban/rural divide is a college/non-college divide. In the past, it was generally thought that as the percentage of college educated persons in a county increase, republicans typically do better. However, as has been noted by some in the literature, this does not hold with Trump (Hull, 2020; Margalit, 2019). As economic insecurity has increased, so has populism—with Trump being one of the best examples of persons who are able to capitalize on the populist message. As a result, college educated voters are beginning to migrate into the democratic party. Although this certainly began before the 2016 election, Trump has definitely increased the rate of migration. Hence, one would expect to find a negative relationship between the percentage of college educated voters and Trump votes. Finally, growth in registration and voter turnout are expected to negatively effect Trump.

A positive relationship is expected *a priori* with the percentage of a county’s population that is white as well as economic factors such as high-income growth and low unemployment rates. These results are expected and well documented in the literature despite the fact that there appears to be some evidence that unemployment rates have a larger impact on voters than income levels or income growth rates (Simon, et al., 1991; Levernier 1993; Mitchell and Willett, 2004). Nevertheless, the Covid-19 pandemic has significantly altered the economic performance of the entire country. Typically, poor economic conditions are blamed on the incumbent party, but it remains to be seen if this general political wisdom holds up in the 2020 election. It is possible that economic conditions will have no effect on the marginal voter’s decision calculus if they perceive that the poor economic conditions are temporary or at least not the fault of Trump. Finally, past support for Trump is expected to have a positive effect on Trump’s ability to attract new voters in the 2020 election.

## Results

Estimation results are shown in Table 5 using weighted least squares to correct for heteroskedasticity and begin with a lean model of past Trump support only on the dependent variable, *TRUMPVOTECHANGEPCT*. Standard errors are shown in parenthesis. Model 2 builds on Model 1 with political variables while Models 3, 4, and 5 add additional demographic and economic variables. Model 6 becomes the model of interest as the Base Covid Model with the addition of the each of the covid independent variables included separately and alone from other covid related variables. To aid the reader in comparing the impact of each covid variable, the covid related coefficient estimates and standard errors are listed in Table 6. Table 6 also includes any other independent variables from the Base Covid Model, e.g. Model 6 from Table 5, that changed in either significance, sign, or size. For example, the Covid Base Model with no covid variables included shows that *TRUMP2016PCT* is positive at a value of 0.115, but not statistically significant. When *USACASESGROW_OCT* is added to the Covid Base Model, there is no statistical significance for the covid variable, but the *TRUMP2016PCT* variable becomes significant and is almost identical in size at 0.147 to its estimate in Model 4 which was 0.146. When the Covid Base Model is estimated with the presence of *USCASESPOP18*, the reader can see from Table 6 that the coefficient estimate for *TRUMP2016PCT* changes size to 0.108 and that *UNEMPLOY2020* decreases in significance from the 5% level to the 10% level while falling in size to -0.845.

**Table 5:** Weighted Least Squares Regression Results for the 2020 Election

RHS variables	Model 1	Model 2	Model 3	Model 4	Model 5	Model 6
Constant	13.560	20.633***	52.809***	59.619***	79.975***	75.293***
	(2.98)	(4.95)	(6.00)	(7.49)	(9.52)	(9.28)
*TRUMP2016PCT*	0.792***	0.837***	0.235***	0.146*	0.114	0.115
	(0.05)	(0.07)	(0.07)	(0.07)	(0.07)	(0.07)
*VISITTRUMP*		-6.373**	-3.00	-1.209	-1.554	-1.781
		(3.24)	(2.65)	(2.47)	(2.44)	(2.39)
*VISITBIDEN*		-1.010	2.016			
		(3.94)	(3.54)			
*VISITPENCE*		-1.381				
		(4.58)				
*VISITHARRIS*		-5.251				
		(6.29)				
*WHITEPOP2020*			0.462***	0.500***	0.572***	0.590***
			(0.05)	(0.05)	(0.05)	(0.05)
*COLLEGE*			-1.627***	-1.774***	-2.482***	-2.69***
			(0.18)	(0.18)	(0.49)	(0.49)
*POPDENSITY*			-0.019***	-0.017***	-0.016***	-0.012***
			(0.004)	(0.003)	(0.003)	(0.003)
*POPDENSITY* ^*2*^			2.14e-06***	2.08e-06***	2.09e-06***	-1.49e-06***
			(5.85e-07)	(5.81e-07)	(5.77E-07)	(3.74e-07)
*POP65PCT2020*			-0.285**	-0.197	-0.118	
			(0.131)	(0.133)	(0.135)	
*INCOME2020*					-0.705***	-0.625***
					(0.20)	(0.20)
*INCOMEGROW*				0.065	0.173	
				(0.15)	(0.15)	
*UNEMPLOY2020*				-1.309***	-1.058**	-1.117**
				(0.46)	(0.46)	(0.46)
INCOME2020* *COLLEGE*					0.023**	0.022**
					(0.01)	(0.01)
Different Covid Variables from Table 6						*XX*
R2	0.35	0.30	0.55	0.56	0.57	0.59
Durbin-Watson	1.79	1.66	1.69	1.71	1.77	1.80
Observations	396	396	396	396	396	396
Note: Dependent Variable is *TRUMPVOTECHANGEPCT* as outlined in Eq. 1. Standard errors are in parenthesis.						
Model 6 is the Covid Base model used to analyze each Covid variable individually.						
* - Significance at the 10% level ; ** - Significance at the 5% level ; *** - Significance at the 1% level						

**Table 6:** Weighted Least Squares Regression Results of Model 6 with Covid Variables

Covid Variable of Interest	Model 6	*TRUMP2016PCT*	*UNEMPLOY2020*	R^2^	DW
*USACASESGROW_OCT*	0.431	0.147*		0.59	1.81
	(0.30)	(0.08)			
*USACASESGROW_SEP*	0.321*	0.163**		0.60	1.83
	(0.16)	(0.08)			
*USCASESPOP18*	0.001***	0.108	-0.845*	0.60	1.85
	(0.0003)	(0.07)	(0.46)		
*USCASESPOP65*	8.63e-05**			0.59	1.82
	(3.70e-05)				
*USCASERATIOPOP18*	3.716***		-0.845*	0.60	1.85
	(1.12)		(0.46)		
*USCASERATIOPOP65*	1.509**			0.59	1.82
	(0.64)				
*USDEATHSGROW_OCT*	0.044	0.159*	-0.731	0.59	1.83
	(0.04)	(0.08)	(0.49)		
*USDEATHSGROW_SEP*	0.121*	0.179**	-0.672	0.61	1.90
	(0.06)	(0.08)	(0.22)		
*USDEATHSPOP18*	0.018*			0.59	1.77
	(0.01)				
*USDEATHSPOP65*	0.003			0.59	1.77
	(0.002)				
*USDEATHRATIOPOP18*	1.658*			0.60	1.85
	(0.899)				
*USDEATHRATIOPOP65*	1.427			0.60	1.85
	(1.042)				
Dependent Variable is *TRUMPVOTECHANGEPCT* as outlined in Eq. 1. Results listed in Table 6 are the Covid Base Model (Model 6) from Table 5 with the addition of each separate covid variable listed alone. Any other independent variables in the Model 6 that changed sign, significance, or size are listed as well. All models had 396 observations. Standard errors are in parenthesis.					
* - Significance at the 10% level; ** - Significance at the 5% level; *** - Significance at the 1% level					

Overall, many of the demographic and economic results are not surprising. *POPDENSITY* has a small but robust and negative effect in all regressions indicating that Trump loses support as a county’s population becomes denser. However, this effect is not strictly linear. When the square of *POPDENSITY* is included within models 3, 4, and 5, it has a small but positive and statistically significant effect signifying that as population density increases, the negative effects on Trump’s reelection begin to decline. For the Covid Base Model, it is negative and statistically significant, but very small in size.

The two other demographic factors were the percent of the county population that has a college degree and race. Generally, for many past election cycles, college educated voters tended to vote Republican, but this trend has been reversing in recent election cycles. This effect in the 2020 election was especially pronounced and statistically significant in all models. In fact, of the variables studied in this analysis, it has the largest effect on the election outcome. The results of *COLLEGE* are robust, negative, and range in size from − 1.63 to -2.69. Similarly, the percent of the county that is white always has a positive effect on Trump’s election outcome. The effect is once again robust, always positive, and statistically significant in every model and values in range from 0.46 to 0.59. However, its values are approximately 1/4th in size compared to the variable *COLLEGE*. Political parties should take note of these results for future elections since both college graduation rates and the percent of the population that is non-white are projected to increase in the future.

The economic variables were of particular interest for this analysis. It is generally expected that voters will punish incumbents for poor economic performance. Nevertheless, it is also possible that there is an asymmetric vote response based upon whether a voter is sophisticated or naïve. Sophisticated voters are able to distinguish short term economic business cycles from trend growth while naïve voters are not. Given this, naïve voters are more likely to reward incumbents when business cycles are positive, while sophisticated voters do not since they are able to differentiate between the cycle and the long-term trend (Maloney & Pickering, 2015). The 2020 election allows a unique test of this hypothesis since the pandemic led to an unusually severe but brief economic downturn in the midst of what was generally an economy experiencing a successful long term growth trend before Covid-19.

The results from the models in Table 5 seem to indicate that voters are indeed naïve and punished the incumbent presidential party. The unemployment rate has a negative and robust effect on Trump’s election outcome in every model. The results are both statistically significant and quite large and range from − 1.06 to -1.3. The size of *UNEMPLOY2020* is especially large when compared to *INCOME2020*. The effect of increasing income is negative and statistically significant and should not be surprising since Trump seems to appeal more to ‘working class’ voters than upper income voters. Surprisingly, *INCOMEGROW* is insignificant in the models shown indicating that voters assign no credit to Trump for income growth over the 2016–2020 timeframe. This confirms earlier research that has shown that voters focus more attention to unemployment rates rather than income measures.

As expected, Trump’s support from the 2016 election represented by *TRUMP2016PCT* plays a part in his support for reelection in 2020. However, this support is not as large as expected, declines, and become insignificant as other independent demographic and economic variables are included in further modeling. Also of interest are the variables on visits to the counties by the candidates. A visit by Trump is negative in every model and significant only in the political model which is Model 2. This also confirms earlier research stating that visits by candidates might increase the mobilization of a party’s voters, but it can also backfire and increase the counter-mobilization of the other candidate’s voters. In this case, it appears that this voter mobilization was, on balance, either not effective or a net loss to Trump.

The Covid-19 results in Table 6 show an interesting phenomenon. Of the twelve different covid variables examined, all are positive in their effect and eight are statistically significant at least at the 10% level. Although there is a large change in the value of the coefficients amongst themselves, the reader will recall that some of the variables, such as *USDEATHRATIOPOP18*, are in a ratio form of county death rates to national death rates while others such as *USCASESPOP18* are the county case rate minus the US case rate. The ratio variables, *USCASERATIOPOP18*, *USCASERATIOPOP65*, *USDEATHRATIOPOP18*, and *USDEATHRATIOPOP65*, have the largest effect on voter behavior. All are significant at the 10% level with the two case-ratio variables significant at the 5% level. For example, a 100- basis point increase in the ratio of county deaths per 100,000 persons relative to national deaths leads to a 166-basis point change in the number of ‘new votes’ that Trump received. When considering the ratio of county level covid case rates to national level covid case rates, the results of a 100-basis point change are even larger—Trump gains 376-basis points of ‘new voters’. That a higher death or case rate in a county relative to the US death or case rate would help Trump is somewhat unexpected. Therefore, additional analysis was undertaken to tease out how increases in covid cases and deaths helped President Trump. The primary way that Trump was helped in the election by high covid cases and deaths was a decrease in voter registration and turnout via the mobilization effect which was concentrated primarily among Biden voters—ostensibly because they were anxious about catching covid during the registration and voting process.

Confirmation of this is shown in Tables 7 and 8. As before, Table 7 contains the base models for understanding how Covid-19 impacted voter registration and turnout with Table 8 showing the impacts of the different covid variables included within the three base models. Interestingly enough, although every county except one had an increase in the number of voters, every county in Arizona, Wisconsin, Michigan, and Pennsylvania also had an increase in the growth of voter turnout relative to the growth of the number of registered voters. Georgia on the other hand saw 147 of its 159 counties with a decline in the growth of voter turnout relative to the growth of registered voters. In other words, voter turnout in Georgia was far below what should have been expected. Because of this, covid’s impact on the growth in the number of voters is estimated separately.

**Table 7:** Weighted Least Squares Regression Results for the 2020 Election on Voter Registration and Turnout

RHS Variables	*REGISTERGROW*	*VOTERGROW* (GA EXCLUDED)	*VOTERGROW* (GA ONLY)
Constant	51.573***	6.241***	12.219***
	(5.70)	(1.17)	(2.83)
*TRUMP2016PCT*	0.364***		
	(0.07)		
*UNEMPLOY2020*	-2.254***	0.693***	-1.514***
	(0.34)	(0.08)	(0.31)
*INCOMEGROW*	-0.652***		
	(0.12)		
*COLLEGE*	0.130		
	(0.12)		
*WHITEPOP2020*	-0.320***		
	(0.05)		
*VISITTRUMP*	-0.129		
	(1.78)		
*VISITBIDEN*	-0.397		
	(1.86)		
*REGISTERGROW*		0.601***	0.427***
		(0.03)	(0.03)
*POP65PCT2020*	-0.304***	0.024	-0.029
	(0.09)	(0.04)	(0.07)
*MICHIGAN*	-4.882***		
	(1.59)		
*WISCONSIN*	-9.521***		
	(1.79)		
Different Covid Variables from Table 8	XX	XX	XX
R2	0.61	0.61	0.61
Durbin-Watson	1.62	2.20	2.24
Observations	396	237	159
Dependent Variable is either *REGISTERGROW* or *VOTERGROW* as outlined in the column. Results listed in Table 7 are the Base Model used to analyze each covid variable individually. Standard errors are in parenthesis.			
* ; ** ; *** ; - Significance at the 10% level; 5% level; and 1% level respectively			

**Table 8:** Weighted Least Squares Regression Results of Table 7 Models with Separate Covid Variables

Covid Variable	*REGISTERGROW*	*VOTERGROW (GA excluded)*	*VOTERGROW (GA ONLY)*
*USCASESGROW_OCT*	-1.197***	0.104	0.796
	(0.215)	(0.077)	(0.641)
*USCASESGROW_SEP*	-0.617***	0.02	0.719
	(0.117)	(0.04)	(0.722)
*USCASESPOP18*	3.65E-05	-0.0001	-0.0002
	(0.0002)	(9.27e-05)	(0.0002)
*USCASESPOP65*	-3.13E-05	-2.36e-05	2.50e-05
	(2.71E-05)	(2.29e-05)	(1.57e-05)
*USCASERATIOPOP18*	0.134	-0.379	-0.689
	(0.854)	(0.341)	(0.721)
*USCASERATIOPOP65*	-0.547	-0.413	0.438
	(0.474)	(0.3999)	(0.275)
*USDEATHSGROW_OCT*	-0.068**	-0.002	0.089
	(0.028)	(0.009)	(0.182)
*USDEATHSGROW_SEP*	-0.122**	-0.015	0.073
	(0.052)	(0.01)	(0.091)
*USDEATHSPOP18*	-0.002	-0.0098**	-0.009**
	(0.007)	(0.003)	(0.004)
*USDEATHSPOP65*	0.001	-0.003***	-0.003***
	(0.001)	(0.0009)	(0.0001)
*USDEATHRATIOPOP18*	-0.167	-0.888**	-0.823**
	(0.666)	(0.344)	(0.324)
*USDEATHRATIOPOP65*	0.577	-1.349***	-1.133***
	(0.771)	(0.403)	(0.368)
Dependent Variable is *REGISTERGROW* or *VOTERGROW*. Results listed in Table 8 are the models from Table 7 with the addition of each separate covid variable listed alone. Standard errors are in parenthesis			
* - Significance at the 10% level; ** - Significance at the 5% level; *** - Significance at the 1% level			

As before, economic and demographic variables have a significant impact on the growth in voter registration. If a county had high rates of income growth, citizens were less interested in registering to vote—presumably because they are content with their economic situation. However, the same cannot be said of high rates of unemployment which tended to decrease voter registration. In addition, nonwhites were more likely to try and register to vote—a demographic which in general hurts President Trump.

When considering registration, the growth rate of the county covid cases had a deleterious and large effect on registration. This can be seen in many ways. For every 1% increase in the percentage of the population that is over age 65, a county experienced a 0.3% decrease in the growth rate of registered voters. Furthermore, the growth rate in the rate of cases from October 1st to election day led to additional and substantial declines in the growth of voters. This was true regardless of the date when a state stopped the voter registration process. Georgia had the earliest cutoff on October 5th for in-person voter registration, while Arizona’s final day was October 15th, Pennsylvania was October 19th, and Michigan and Wisconsin were election day itself. Since Michigan and Wisconsin had an extended period of voter registration relative to the three other states, their fixed effect is shown in Table 7. Both states experienced a large and negative decline in voter registration that was statistically significant relative to what should have been expected in a normal election cycle.

This decrease in the number of new persons registering to vote translates into a decrease in the growth of voter turnout. Table 7 has the base models for *VOTERGROW* with Georgia excluded and Georgia estimated on its own. In Georgia, every 100-basis point increase in registration growth only leads to a 42.7 basis point increase in voter turnout. This is about 30% smaller than in the other four states. Furthermore, unemployment in Georgia has a large and negative impact on voter turnout while in the other four states, higher unemployment leads to an increase in voter turnout. As with voter registration growth, Covid-19 has a significant and negative effect on turnout. A 100-basis point increase in the ratio of the county death rates relative to the national death rates decreases the growth rate of voter turnout by 113 to 135 basis points depending upon the state in question.

To see this effect with real numbers, a dynamic simulation was run that would produce a 10% decline in deaths on election day in each county. The corresponding decrease in cases needed to reduce deaths by 10% was also estimated to determine the change in registered voters, voter turnout, and voter preference.^4^ Results indicated that the size of Trump’s loss in each state would have grown, but by varying amounts. For example, in Arizona, Trump would have lost by nearly 13,000 votes while in Georgia his loss would have extended to a little over 15,000 votes. Similarly, Michigan and Pennsylvania would have seen loss extensions of 2,200 and 2,500 votes respectively. In Wisconsin, Trump’s loss would have been extended by less than 100 votes.

## Conclusion

The 2020 Presidential election was somewhat unique in recent US history in that it occurred during a period of economic uncertainty, a global pandemic, and between two major political party candidates who had relatively high negative ratings by voters. Furthermore, the election was decided by very razor thin margins within 3 states and very small margins in 2 others. Under normal circumstances, the performance of the economy will impact the re-election chances of a Presidential candidate. The 2020 election was no exception with economic factors playing a large role in determining how people voted. In addition, the unemployment rate appears to have had a larger effect on voter’s decision calculus than income growth. In this respect, this research has added more evidence to the question of whether people vote with their pocketbooks, and if so, which economic issue is larger in their minds—a job or income. It appears to be the former.

However, running alongside the economic factors were once-in-a-century public health factors. This research shows that the effect of Covid-19, although small in size, was large enough to affect statewide election results in 5 different states and make the election much closer than it otherwise would have been. Covid-19 decreased both voter registration and voter turnout—two factors which played in President Trump’s favor even though he did not win any of these states. Thanks to Covid-19, the mobilization effect dominated the obituary effect and the alteration effect and this mobilization effect primarily was experienced by citizens who would have cast a vote for Biden. In other words, without Covid-19, President Trump would have lost by even larger margins that he did. This result is confirmed under a simulation of only a 10% decrease in the number of Covid-19 deaths in each county.

Finally, the results presented here have raised several questions for future research. Questions in past research have examined whether voters are sophisticated or naïve vis-à-vis economic growth conditions and the assigning of ‘blame’ or ‘credit’ to incumbents. The research presented here seems to support the naïve hypothesis whereby voters blame incumbents on economic conditions that deviate outside of normal growth patterns. The unique and rapid whiplash nature of the economy, and the cause of this whiplash, in 2020 provides an exceptional opportunity to study this hypothesis in more detail. A second research question is to the size and direction of blame that Trump received for Covid-19 across the country and to its effect on voting behavior in other states—especially ones that are considered ‘safe’ by either party. Additional research also needs to be spent on understanding the relationship between registration and voting in Georgia in greater detail. This analysis has shown that there was a disconnect between these two behaviors that did not exist in the other four states. Finally, this research did not consider what role stimulus checks and extended unemployment compensation may or may not have played in altering voter’s decisions not only within these 5 states, but across the entire country. Research on the key extensions of this analysis is already underway.

## Data Availability

All data used within this manuscript is publicly available free of charge.

## Footnotes

1 The full poll results are available at http://cdn.cnn.com/cnn/2019/images/12/20/rel14c.-.economy,.general.election.pdf and includes other questions on economic conditions, international trade agreements, and favorability ratings of Democratic Presidential candidates.

2 https://www.usatoday.com/story/news/politics/elections/2019/12/16/trump-impeachment-2020-election-leads-democratic-rivals/2663659001/.

3 It is recognized that not everyone who voted for Trump in 2016 voted for Trump in 2020 and visa-versa. It is also recognized that not everyone who voted for Clinton in 2016 voted for Biden in 2020 and visa-versa. It is recognized that voters can, and sometimes do, alter the party or person that they voted for in past elections. This model is not claiming that every voter in 2020 who voted in 2016 voted in an identical way. The model is simply looking at the ‘marginal 2020 voters’ and attempting to understand what factors influenced their vote.

4 There is some evidence that deaths followed quickly after initial Covid-19 case identification. Raymond (2021) found that the median lag was 8.1 days.
